# Finite-time robust motion control of intelligent vehicles based on neural approximation and disturbance estimation

**DOI:** 10.1371/journal.pone.0362314

**Published:** 2026-11-06

**Authors:** Lijuan Zhang

**Affiliations:** Nantong Open University, Jiangsu, Nantong, China; Guangdong University of Technology, CHINA

## Abstract

This paper addresses the inherent trade off between rapid convergence and high‑precision disturbance rejection in intelligent vehicle motion control under severe nonlinearities, parametric uncertainties, and time varying external perturbations. A composite finite‑time robust control strategy is proposed by synergistically integrating a nonsingular fast terminal sliding mode (NFTSM) surface, radial basis function neural networks (RBFNN), and a finite time disturbance observer (FTDO). The NFTSM guarantees singularity free finite time convergence of the tracking errors. The RBFNN approximates the slowly varying unknown dynamics, while the FTDO compensates online for the neural approximation residual and fast varying disturbances in a cascaded “coarse to fine” manner. Lyapunov-based Extensive co‑simulations with MATLAB/Simulink, conducted under double lane change and sinusoidal path scenarios with simultaneous road friction drops and stochastic lateral wind gusts, demonstrate that the proposed framework significantly outperforms conventional nonsingular terminal sliding mode control and disturbance observer based sliding mode control in terms of tracking accuracy, convergence speed, and chattering suppression.

## 1 Introduction

The rapid advancement of intelligent vehicles complex cyber-physical systems integrating perception, decision-making, and motion execution has rendered motion control a central challenge in autonomous driving research [1–3]. The core task of accurately tracking a desired path while preserving stability is severely complicated by strongly nonlinear tire [4], road interactions [5], parametric uncertainties [6], unmodeled dynamics [7], and time-varying external disturbances such as friction variations and wind gusts [8]. These factors collectively impose stringent requirements: the control system must deliver fast transient response, high steady-state accuracy, and robust performance against diverse uncertainties, all while respecting actuator limits and ride comfort.

In the past few decades, a lot of research work has been devoted to vehicle motion control, resulting in a wealth of control methods [9–11]. Common ones are PID control [12], adaptive control [13], robust control [14] and intelligent control [15]. In [16], Zhang et al. proposed a reinforcement learning-based intelligent control strategy for the air conditioning system of electric vehicles, aiming to optimize energy consumption while maintaining cabin thermal comfort. Fei et al. [17] addressed the longitudinal tracking challenge of intelligent vehicles by designing a control strategy that explicitly compensates for road gradient and load disturbances, thereby enhancing robustness against external perturbations. In [18], Zou and Chen proposed a metaheuristic optimization framework that simultaneously optimizes flight control and power conversion for fuel cell-powered quadrotors, aiming to enhance overall system efficiency and flight stability under dynamic operating conditions. In [19], Chaturvedi et al. proposed an AI and IoT-integrated adaptive control framework for electric vehicles, which leverages real-time sensor data and intelligent decision-making to enhance vehicle performance, energy efficiency, and overall system reliability. In [20], Liu et al. proposed an intelligent cooperative tracking control strategy for both longitudinal and lateral dynamics of intelligent vehicles, which integrates feed forward compensation with error model-based feedback to enhance trajectory tracking accuracy and driving stability.

Sliding mode control (SMC) has long been recognized as an effective tool for robust control of nonlinear systems due to its inherent invariance to matched uncertainties and disturbances [21–23]. In [24], Demim et al. proposed an integrated SMC framework combined with a high-gain observer for unmanned aerial vehicle (UAV) navigation, which enhances trajectory tracking and state estimation robustness under uncertain and noisy operating conditions. In [25], Luo and Wang proposed a distributed formation control strategy for multi-quadrotor UAV systems, which employs fractional-order robust sliding mode trajectory tracking to achieve precise formation maintenance and enhanced disturbance rejection under complex flight conditions. Baek et al. [26] addressed the trajectory tracking control challenge of robot manipulators by developing an adaptive SMC strategy that combines the robustness of SMC with adaptive gain tuning to handle parameter variations and external disturbances. In [27], Alqaisi et al. proposed a feedback linearization-based control strategy integrated with SMC for four-degree-of-freedom robot manipulators, which effectively handles system nonlinearities and uncertainties to achieve precise trajectory tracking. In addition, Liang [28] addressed the trajectory tracking control challenge of robotic systems by developing a hybrid control framework that combines neural networks’ adaptive approximation capability with SMC’s robustness, achieving accurate and stable tracking under model uncertainties. However, conventional linear sliding mode control only guarantees asymptotic convergence, which may be insufficient for safety-critical maneuvers requiring rapid response. In order to overcome this limitation, terminal sliding mode control (TSM) is introduced to achieve finite time convergence by combining nonlinear terms with sliding surfaces [29]. Nonsingular fast terminal sliding mode (NFTSM) further solves the inherent singularity problem of traditional terminal sliding mode, and accelerates the convergence in the region near and far from the origin [30]. Recently, advanced control schemes have been developed for robotic systems with complex actuator dynamics and communication constraints. Ma et al. [31] proposed a hyperchaotic privacy-preserving cooperative adaptive control method for multimotor systems, whose novelty lies in combining hyperchaotic encryption with cooperative adaptive control to achieve secure and coordinated tracking. In [32], Ma et al. developed an actor–critic-based prescribed performance optimal control strategy for flexible-joint robots with input delay, innovatively integrating prescribed performance, optimal control, and actor–critic learning to handle input delay while guaranteeing transient and steady-state performance. These works focus on different plant dynamics and control objectives from intelligent vehicle motion control.

Disturbance observer (DOB) techniques offer an alternative paradigm by explicitly estimating and compensating for unknown disturbances and model uncertainties [33–35]. The nonlinear disturbance observer (NDO) and its variants have been widely adopted in vehicle systems. More recently, finite-time disturbance observers (FTDO) have been developed to achieve accurate estimation within a bounded time interval [36], enabling tighter integration with finite-time control frameworks. Nevertheless, disturbance observers face inherent limitations. When the system contains severe nonlinearities and time-varying parameters, the lumped disturbance to be estimated becomes highly complex, making it difficult to achieve both fast convergence and high estimation accuracy simultaneously. Neural networks, particularly radial basis function neural networks (RBFNN) [37], possess universal approximation capabilities and have been extensively employed to handle unknown nonlinearities in dynamical systems. However, most existing neural network-based controllers provide only asymptotic stability, lacking the deterministic convergence time guarantees essential for safety-critical applications.

To bridge these gaps, this paper proposes a composite finite-time robust motion control strategy integrating NFTSM, RBFNN, and FTDO within a unified approximation–estimation–control framework, featuring a cascaded coarse-to-fine estimator where the RBFNN approximates slow unknown dynamics and the FTDO compensates for residuals and fast disturbances. The main contributions are:

1. A triple-layer architecture that organically combines NFTSM, RBFNN, and FTDO, systematically resolving the conflict between fast convergence and high-precision compensation.
2. A two-stage cascaded estimator exploiting the complementary strengths of neural approximation and finite-time disturbance rejection, with RBFNN providing coarse estimation and FTDO refining residuals.
3. A rigorous Lyapunov-based stability analysis proving finite-time boundedness of all closed-loop signals and providing explicit convergence-time bounds for parameter tuning.
4. Comprehensive CarSim–Simulink validations under double lane change and sinusoidal scenarios with simultaneous friction drops and wind gusts, including comparative and ablation studies to confirm individual and synergistic effects.

The remainder of this paper is organized as follows. Sect 2 establishes the vehicle dynamics model and derives the path tracking error dynamics. Sect 3 presents the proposed composite control framework, detailing the nonsingular fast terminal sliding mode surface, the RBF neural network approximator, the finite-time disturbance observer, the complete control law, and the closed‑loop stability analysis. Sect 4 provides simulation results and ablation studies to demonstrate the effectiveness of the proposed method. Finally, Sect 5 concludes the paper and discusses future research directions.

## 2 Vehicle dynamics modeling and problem formulation

This section establishes a comprehensive mathematical model for the lateral motion control of an intelligent vehicle. A planar single-track (bicycle) model is adopted to capture the essential lateral and yaw dynamics while maintaining analytical tractability for control design. The model incorporates nonlinear tire force characteristics, parameter uncertainties, and external disturbances, which collectively constitute the basis for the subsequent finite-time robust control synthesis. All variables are defined upon their first appearance, and standard assumptions are stated to render the problem well-posed.

### 2.1 Notation and coordinate systems

Before proceeding to the dynamic equations, we define the coordinate systems used throughout this paper. As illustrated in Fig 1, two coordinate frames are employed:

- A global inertial frame ${ℱ}_{G}=\{O_{G},X_{G},Y_{G},Z_{G}\}$ fixed to the ground, with the $X_{G}$-$Y_{G}$ plane being the horizontal plane of motion.
- A body-fixed frame ${ℱ}_{B}=\{O_{B},x,y,z\}$ attached to the vehicle at its center of gravity (CG), with the *x*-axis pointing forward along the vehicle’s longitudinal direction, the *y*-axis pointing to the driver’s left, and the *z*-axis pointing upward following the right-hand rule.

**Fig 1:**
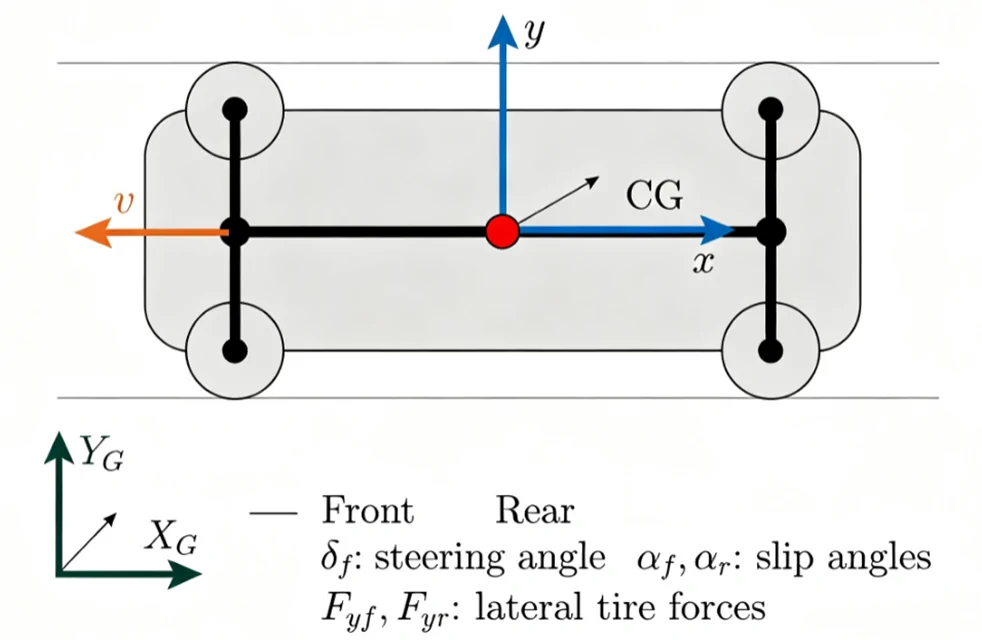
Schematic diagram of the single-track vehicle model.

### 2.2 Vehicle kinematics and dynamics

The following standard assumptions are imposed to derive the vehicle model.

**Assumption 1:** The vehicle is modeled as a rigid body with a fixed mass distribution. Pitch, roll, and vertical motions are neglected, as the primary focus is on lateral motion control in the horizontal plane.

**Assumption 2:** The longitudinal velocity $v_{x}$ is treated as a bounded, time-varying parameter that is measurable but not directly controlled. This separation is justified in scenarios where longitudinal motion is governed by a separate driver or upper-level controller.

Under these assumptions, the vehicle’s lateral and yaw motions are described by Newton’s second law applied to the CG:


$$\begin{array}{cc} m({\dot{v}}_{y}+v_{x}\dot{\psi }) & =\sum F_{y}=F_{yf}+F_{yr}+d_{y}(t), \end{array}$$ (1)

$$\begin{array}{cc} I_{z}\ddot{\psi } & =\sum M_{z}=l_{f}F_{yf}-l_{r}F_{yr}+d_{\psi }(t), \end{array}$$ (2)

where the variables and parameters are defined in Table 1.

**Table 1:** Vehicle state variables and physical parameters.

Symbol	Unit	Description
*m*	kg	Total vehicle mass
$I_{z}$	kg·m^2^	Yaw moment of inertia about the vertical axis
$l_{f}$	m	Distance from CG to front axle
$l_{r}$	m	Distance from CG to rear axle
$v_{x}$	m/s	Longitudinal velocity at CG
$v_{y}$	m/s	Lateral velocity at CG
ψ	rad	Yaw angle (orientation of the vehicle relative to the global frame)
$\dot{\psi }$	rad/s	Yaw rate
$F_{yf}$	N	Total lateral tire force at the front axle
$F_{yr}$	N	Total lateral tire force at the rear axle
$d_{y}(t)$	N	Lumped external disturbance in the lateral direction
$d_{\psi }(t)$	N·m	Lumped external disturbance moment about the vertical axis

### 2.3 Tire force modeling

The lateral tire forces $F_{yf}$ and $F_{yr}$ are functions of the tire slip angles $\alpha_{f}$ and $\alpha_{r}$, respectively. The slip angles are defined as the angle between the tire’s actual velocity vector and its heading direction, as illustrated in Fig. 2. For small slip angles, the tire forces can be linearized as:


$$\begin{array}{c} F_{yf}=C_{f}\alpha_{f},\;F_{yr}=C_{r}\alpha_{r}, \end{array}$$ (3)

where $C_{f}>0$ and $C_{r}>0$ are the front and rear cornering stiffness coefficients.

**Fig 2:**
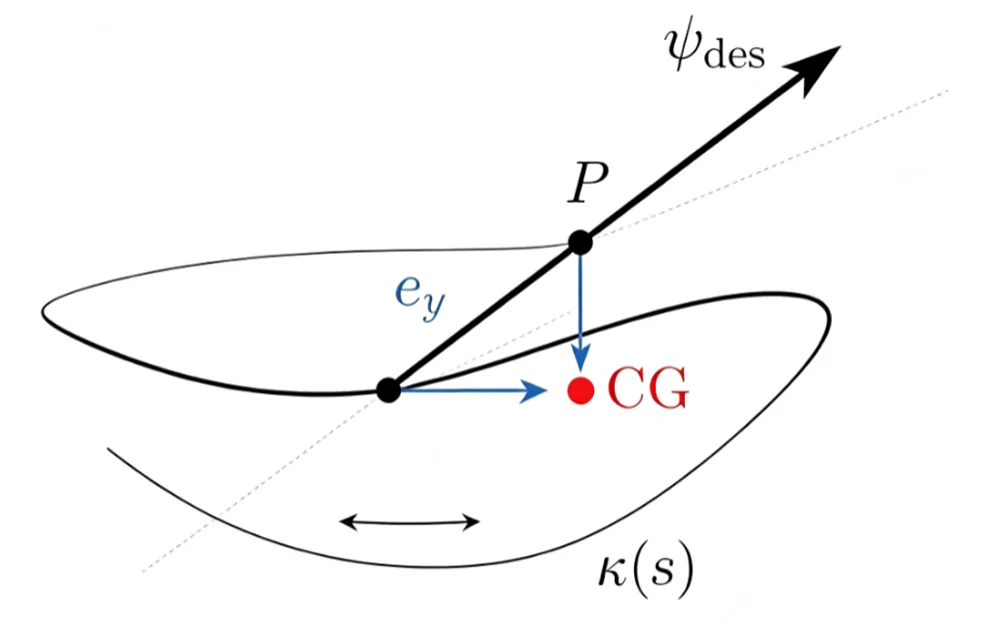
Geometric interpretation of the path tracking error variables.

**Assumption 3:** The tire slip angles remain sufficiently small such that the linear tire model in (3) holds with acceptable accuracy. Nonlinear tire characteristics are treated as unmodeled dynamics and lumped into the disturbance terms.

The kinematic relations for the slip angles are derived from the velocity components at each axle:


$$\begin{array}{cc} \alpha_{f} & =\delta_{f}-\frac{v_{y}+l_{f}\dot{\psi }}{v_{x}}, \end{array}$$ (4)

$$\begin{array}{cc} \alpha_{r} & =-\frac{v_{y}-l_{r}\dot{\psi }}{v_{x}}, \end{array}$$ (5)

where $\delta_{f}$ is the front wheel steering angle, which serves as the control input to the system. Substituting (4) and (5) into (3), and then into (1)–(2), yields the state-space representation:


$$\begin{array}{cc} {\dot{v}}_{y} & =\frac{C_{f}+C_{r}}{mv_{x}}v_{y}+\left(\frac{l_{f}C_{f}-l_{r}C_{r}}{mv_{x}}-v_{x}\right)\dot{\psi }-\frac{C_{f}}{m}\delta_{f}+\frac{d_{y}}{m}, \end{array}$$ (6)

$$\begin{array}{cc} \ddot{\psi } & =\frac{l_{f}C_{f}-l_{r}C_{r}}{I_{z}v_{x}}v_{y}+\frac{l_{f}^{2}C_{f}+l_{r}^{2}C_{r}}{I_{z}v_{x}}\dot{\psi }-\frac{l_{f}C_{f}}{I_{z}}\delta_{f}+\frac{d_{\psi }}{I_{z}}. \end{array}$$ (7)

**Remark 1:** The model (6)–(7) is linear time-varying due to the dependence on $v_{x}(t)$. For control design, it is often converted into error coordinates relative to a desired path, as shown in the next subsection.

### 2.4 Path tracking error dynamics

To formulate the tracking control problem, we define the errors relative to a desired path 𝒫 parameterized by arc length *s*. The desired path is assumed to be sufficiently smooth and is described by the following quantities:

- *y*_des_(*s*): desired lateral displacement in the global frame.
- $\psi_{\text{des}}(s)$: desired yaw angle (tangent angle of the path).
- $\kappa (s)=d\psi_{\text{des}}/ds$: path curvature.

The lateral tracking error $e_{y}$ and the yaw angle error $e_{\psi }$ are defined as:


$$\begin{array}{cc} e_{y} & ≜y-y_{\text{des}}, \end{array}$$ (8)

$$\begin{array}{cc} e_{\psi } & ≜\psi -\psi_{\text{des}}. \end{array}$$ (9)

Under the assumption that the path curvature varies slowly (i.e., $\dot{\kappa }\approx 0$), the time derivatives of the desired quantities satisfy:


$$\begin{array}{cc} {\dot{y}}_{\text{des}} & =v_{x}\sin e_{\psi }\approx v_{x}e_{\psi }, \end{array}$$ (10)

$$\begin{array}{cc} {\dot{\psi }}_{\text{des}} & =v_{x}\kappa ,\;{\ddot{\psi }}_{\text{des}}=v_{x}\dot{\kappa }\approx 0. \end{array}$$ (11)

Using (6)–(7), the error dynamics can be expressed in compact state-space form as:


$$\begin{array}{c} {}[\begin{array}{c} {\dot{e}}_{y}\\ {\ddot{e}}_{y}\\ {\dot{e}}_{\psi }\\ {\ddot{e}}_{\psi } \end{array}]=A(v_{x})[\begin{array}{c} e_{y}\\ {\dot{e}}_{y}\\ e_{\psi }\\ {\dot{e}}_{\psi } \end{array}]+B\delta_{f}+D(t), \end{array}$$ (12)

where the state matrix $A(v_{x})\in {\mathbb{R}}^{4\times 4}$ and input matrix $B\in {\mathbb{R}}^{4\times 1}$ are given by:


$$\begin{array}{cc} A(v_{x}) & =[\begin{array}{cccc} 0 & 1 & 0 & 0\\ 0 & -\frac{C_{f}+C_{r}}{mv_{x}} & \frac{C_{f}+C_{r}}{m} & \frac{l_{f}C_{f}-l_{r}C_{r}}{mv_{x}}\\ 0 & 0 & 0 & 1\\ 0 & -\frac{l_{f}C_{f}-l_{r}C_{r}}{I_{z}v_{x}} & \frac{l_{f}C_{f}-l_{r}C_{r}}{I_{z}} & -\frac{l_{f}^{2}C_{f}+l_{r}^{2}C_{r}}{I_{z}v_{x}} \end{array}], \end{array}$$ (13)

$$\begin{array}{cc} B & =[\begin{array}{c} 0\\ \frac{C_{f}}{m}\\ 0\\ \frac{l_{f}C_{f}}{I_{z}} \end{array}]. \end{array}$$ (14)

The term $D(t)\in {\mathbb{R}}^{4}$ is the lumped disturbance vector:


$$\begin{array}{c} D(t)=[\begin{array}{c} 0\\ \Delta_{y}(t)\\ 0\\ \Delta_{\psi }(t) \end{array}], \end{array}$$ (15)

where $\Delta_{y}(t)$ and $\Delta_{\psi }(t)$ represent the combined effects of:

- parametric uncertainties in *m*, $I_{z}$, $C_{f}$, $C_{r}$, $l_{f}$, $l_{r}$;
- unmodeled nonlinear tire dynamics (e.g., force saturation);
- external disturbances such as side wind gusts and road irregularities;
- coupling effects from longitudinal motion when $v_{x}$ varies.

**Remark 2:** The disturbances $\Delta_{y}(t)$ and $\Delta_{\psi }(t)$ are not necessarily matched, meaning they enter the system through different channels than the control input. This poses challenges for standard sliding mode control, which typically handles only matched uncertainties.

### 2.5 Control-oriented reduced dynamics

For controller design, it is convenient to focus on the lateral error subsystem, as the yaw error is naturally coupled through the kinematic relation. From (12), the following second-order differential equation for the lateral error can be isolated:


$$\begin{array}{c} {\ddot{e}}_{y}=f(e_{y},{\dot{e}}_{y},e_{\psi },{\dot{e}}_{\psi },v_{x})+b\delta_{f}+\Delta (t), \end{array}$$ (16)

where:


$$\begin{array}{cc} f(\cdot ) & =-\frac{C_{f}+C_{r}}{mv_{x}}{\dot{e}}_{y}+\frac{C_{f}+C_{r}}{m}e_{\psi }+\frac{l_{f}C_{f}-l_{r}C_{r}}{mv_{x}}{\dot{e}}_{\psi }, \end{array}$$ (17)

$$\begin{array}{cc} b & =\frac{C_{f}}{m}>0, \end{array}$$ (18)

$$\begin{array}{cc} \Delta (t) & =\Delta_{y}(t). \end{array}$$ (19)

**Assumption 4:** The control gain $b=C_{f}/m$ is strictly positive and bounded away from zero, i.e., there exists a constant $\underset{―}{b}>0$ such that $b\geq \underset{―}{b}$. Since $C_{f}>0$ and *m* > 0, this condition is naturally satisfied with $\underset{―}{b}=C_{f}/m$.

The system (16) is in a form amenable to sliding mode control design, with f(·) representing the known nominal dynamics and Δ(t) the unknown lumped disturbance to be estimated and compensated.

**Assumption 5:** The lumped disturbance Δ(t) and its first time derivative $\dot{\Delta }(t)$ are bounded on [0,∞), i.e., there exist positive constants $\bar{\Delta }$ and ${\bar{\Delta }}^{\left(1\right)}$ such that:


$$\begin{array}{c} |\Delta (t)|\leq \bar{\Delta },\;|\dot{\Delta }(t)|\leq {\bar{\Delta }}^{\left(1\right)},\;\forall t\geq 0. \end{array}$$ (20)

**Assumption 6:** The full state vector $[e_{y},{\dot{e}}_{y},e_{\psi },{\dot{e}}_{\psi }{]}^{⊤}$ is measurable. In practice, these quantities are obtained from onboard sensors combined with appropriate filtering.

**Assumption 7:** The desired path is twice continuously differentiable with bounded curvature: $\kappa (s)\leq \kappa_{\max}$ for all *s*, ensuring that the desired yaw rate ${\dot{\psi }}_{\text{des}}=v_{x}\kappa$ remains bounded.

**Remark 3:** Assumption 5 does not require the bounds to be known exactly; they are used only for theoretical analysis. The proposed finite-time disturbance observer will estimate Δ(t) online, and the sliding mode term will handle the estimation error.

**Remark 4:** The reduced dynamics (16) will serve as the basis for the neural network approximation and finite-time disturbance observer design in the next section. The unknown function f(·) will be approximated online by an RBF neural network, while the residual disturbance Δ(t) will be reconstructed by the FTDO.

## 3 Finite-time robust control design via neural approximation and disturbance estimation

This section presents the detailed design of the proposed composite finite-time robust control strategy. The overall architecture, depicted in Fig 3, consists of three hierarchically integrated modules: (i) a nonsingular fast terminal sliding mode (NFTSM) surface that establishes the finite-time convergence framework; (ii) a RBFNN that approximates the slowly varying unknown dynamics; and (iii) a finite-time disturbance observer (FTDO) that compensates for the neural approximation residual and fast-varying external perturbations in a cascaded manner. The synergy among these three components yields a “coarse-to-fine” estimation-compensation-control paradigm that resolves the inherent trade-off between rapid convergence and high-precision disturbance rejection.

**Fig 3:**
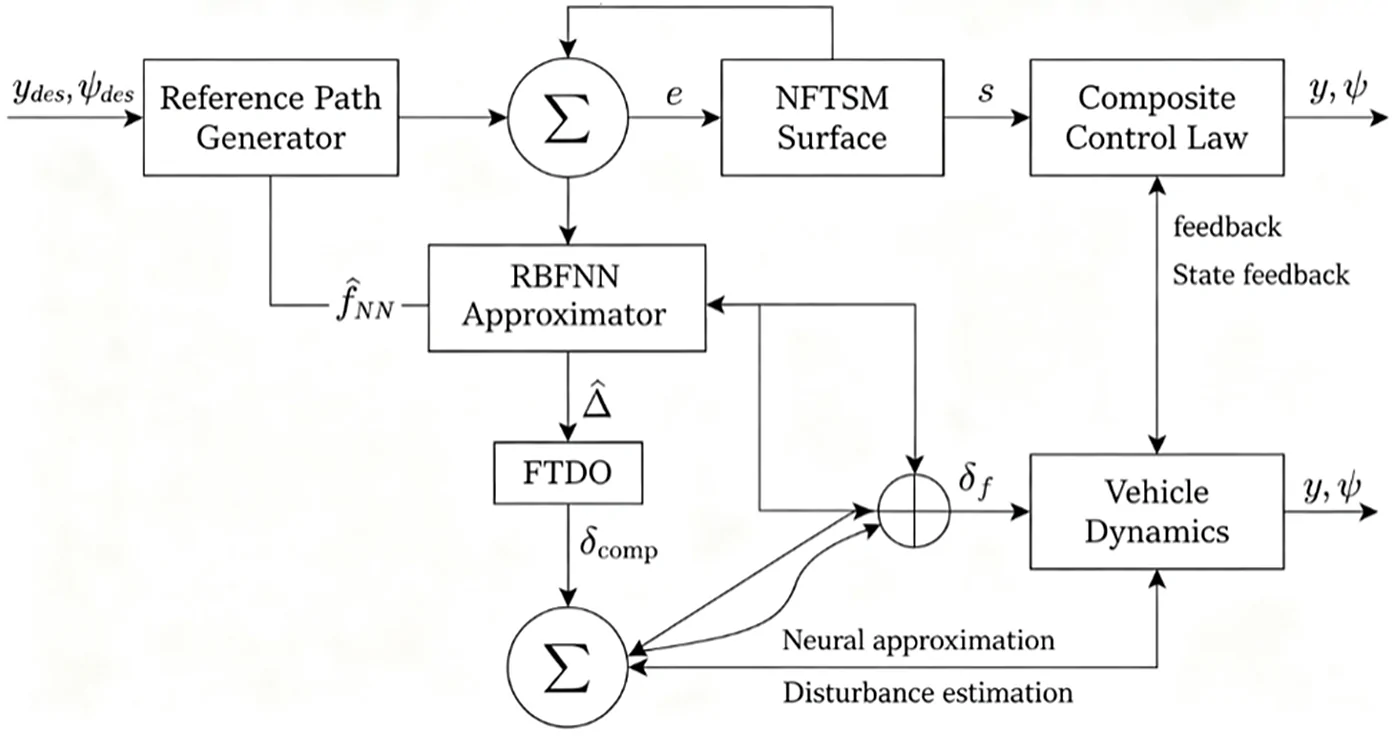
Geometric interpretation of the path tracking error variables.

### 3.1 Nonsingular fast terminal sliding mode surface design

The sliding mode surface is the cornerstone of the proposed control framework, as it determines the convergence behavior of the tracking errors once the sliding mode is established. To achieve finite-time convergence while avoiding the singularity problem inherent in conventional terminal sliding modes, we adopt the nonsingular fast terminal sliding mode (NFTSM) surface.

Consider the reduced lateral error dynamics derived in (16):


$$\begin{array}{c} {\ddot{e}}_{y}=f(e_{y},{\dot{e}}_{y},e_{\psi },{\dot{e}}_{\psi },v_{x})+b(v_{x})\delta_{f}+\Delta (t), \end{array}$$ (21)

where $e_{y}$ is the lateral tracking error, $\delta_{f}$ is the steering angle input, f(·) represents the known nominal dynamics, $b(v_{x})>0$ is the control gain, and Δ(t) is the lumped unknown disturbance.

Define the NFTSM surface s∈ℝ as:


$$\begin{array}{c} s={\dot{e}}_{y}+\alpha e_{y}+\beta \cdot {\text{sig}}^{p/q}(e_{y}), \end{array}$$ (22)

where α>0 and β>0 are positive design constants, *p* and *q* are positive odd integers satisfying 1 < *p*/*q* < 2, and the function ${\text{sig}}^{r}(\cdot )$ is defined for any *r* > 0 as:


$$\begin{array}{c} {\text{sig}}^{r}(x)=|x{|}^{r}\text{sgn}(x),\;x\in \mathbb{R}. \end{array}$$ (23)

**Remark 5:** The choice of p/q∈(1,2) ensures that when the system state is far from the origin ($|e_{y}|\gg 1$), the term $\beta \cdot {\text{sig}}^{p/q}(e_{y})$ dominates, providing fast convergence. When the state is near the origin, the linear term $\alpha e_{y}$ takes over, preventing the control signal from becoming singular.

The following theorem characterizes the convergence behavior of the system when confined to the sliding surface.

**Theorem 1:** Consider the error dynamics in (16) with Δ(t)=0 and suppose the NFTSM surface in (22) is designed with α>0, β>0, and *p*, *q* positive odd integers with 1 < *p*/*q* < 2. If the system state reaches the sliding surface *s* = 0 at time $t_{s}$, then the tracking error $e_{y}(t)$ converges to zero in finite time, and the convergence time $T_{s}$ satisfies:


$$\begin{array}{c} T_{s}\leq \frac{q}{\alpha (q-p)}\ln\left(1+\frac{\alpha }{\beta }|e_{y}(t_{s}){|}^{(q-p)/q}\right). \end{array}$$ (24)

**Proof 1:** On the sliding surface *s* = 0, we have from (22):


$$\begin{array}{c} {\dot{e}}_{y}=-\alpha e_{y}-\beta \cdot {\text{sig}}^{p/q}(e_{y}). \end{array}$$ (25)

Consider the Lyapunov function candidate $V_{1}=\frac{1}{2}e_{y}^{2}$. Its time derivative along (25) is:


$$\begin{array}{cc} {\dot{V}}_{1} & =e_{y}{\dot{e}}_{y}=-\alpha e_{y}^{2}-\beta e_{y}\cdot {\text{sig}}^{p/q}(e_{y})\\ & =-\alpha e_{y}^{2}-\beta |e_{y}{|}^{p/q+1}. \end{array}$$ (26)

Define $z=\sqrt{2V_{1}}=|e_{y}|$. Then $V_{1}=\frac{1}{2}z^{2}$ and ${\dot{V}}_{1}=z\dot{z}$. Substituting $e_{y}^{2}=z^{2}$ and $|e_{y}|=z$ into (26) yields


$$\begin{array}{c} z\dot{z}=-\alpha z^{2}-\beta z^{p/q+1}. \end{array}$$ (27)

Since *z* > 0 for $e_{y}\neq 0$, dividing both sides by *z* gives


$$\begin{array}{c} \dot{z}=-\alpha z-\beta z^{p/q}. \end{array}$$ (28)

Solving this differential equation yields the upper bound in (24), which confirms finite-time convergence. The singularity-free property follows from the fact that the term ${\dot{e}}_{y}$ appears linearly in the surface definition, and the resulting control law does not contain negative powers of $e_{y}$. This completes the proof.

**Remark 6:** The convergence time $T_{s}$ is monotonically decreasing with respect to α and β, meaning that larger gains yield faster convergence. However, excessively large gains may excite unmodeled high-frequency dynamics and increase control effort. The trade-off between convergence speed and control smoothness must be carefully balanced in practice.

### 3.2 Radial basis function neural network approximator

The term f(·) in (16) represents the known nominal dynamics derived from the vehicle model. However, in practice, the actual dynamics deviate from the nominal model due to parameter uncertainties and unmodeled nonlinearities. To address this, we employ a RBFNN to approximate the unknown part of the system dynamics online.

The unknown nonlinear function *f*_unk_(*x*) is approximated by the RBFNN as:


$$\begin{array}{c} f_{\text{NN}}(x)=W^{*⊤}\Phi (x)+\varepsilon (x), \end{array}$$ (29)

where:

- $x\in {\mathbb{R}}^{n}$ is the input vector to the neural network;
- $W^{*}\in {\mathbb{R}}^{N}$ is the ideal weight vector that minimizes the approximation error;
- $\Phi (x)=[\phi_{1}(x),\phi_{2}(x),...,\phi_{N}(x){]}^{⊤}\in {\mathbb{R}}^{N}$ is the vector of radial basis functions;
- ε(x) is the network reconstruction error, bounded by $\bar{\varepsilon }>0$.

The input vector *x* is selected to include the relevant states that characterize the vehicle’s dynamic behavior:


$$\begin{array}{c} x=[e_{y},{\dot{e}}_{y},e_{\psi },{\dot{e}}_{\psi },v_{x},{\dot{\psi }}_{\text{ref}}{]}^{⊤}. \end{array}$$ (30)

The radial basis functions $\phi_{i}(x)$ are chosen as Gaussian functions:


$$\begin{array}{c} \phi_{i}(x)=\exp\left(-\frac{\|x-c_{i}{\|}^{2}}{2\sigma_{i}^{2}}\right),\;i=1,2,...,N, \end{array}$$ (31)

where $c_{i}\in {\mathbb{R}}^{n}$ and $\sigma_{i}>0$ are the center and width of the *i*-th basis function, respectively.

**Assumption 8:** The ideal weight vector $W^{*}$ is bounded, i.e., there exists a known constant $W_{M}>0$ such that $\|W^{*}\|\leq W_{M}$. The reconstruction error ε(x) is uniformly bounded by $\bar{\varepsilon }>0$ over the compact set $\Omega_{x}\subset {\mathbb{R}}^{n}$ that contains all possible system trajectories.

Let $\hat{W}$ denote the estimate of the ideal weight vector $W^{*}$. The online approximation of the unknown dynamics is then given by:


$$\begin{array}{c} {\hat{f}}_{\text{NN}}(x)={\hat{W}}^{⊤}\Phi (x). \end{array}$$ (32)

The weight adaptation law is designed using the Lyapunov-based approach to guarantee closed-loop stability. Specifically, the adaptive law is chosen as:


$$\begin{array}{c} \dot{\hat{W}}=\Gamma \Phi (x)s, \end{array}$$ (33)

where $\Gamma =\Gamma^{⊤}>0$ is the positive-definite adaptation gain matrix, and *s* is the sliding variable defined in (22). This update law is derived from the Lyapunov analysis presented in Sect 3.5.

**Remark 7:** The update law (33) is of gradient-descent type and drives the weight estimates toward values that minimize the sliding variable *s*. The use of *s* as the error signal ensures that the neural network learns in a manner that directly benefits the control objective, rather than pursuing standalone function approximation accuracy.

**Remark 8:** The separation of the unknown dynamics into a slowly varying component and a fast-varying/residual component is not a strict assumption on the external disturbance. It is motivated by the time-scale separation between the adaptation bandwidth of the RBFNN and the finite-time convergence rate of the FTDO. Mathematically, any bounded unknown function *f*_unk_(*x*,*t*) can be decomposed as


$$\begin{array}{c} f_{\text{unk}}(x,t)=f_{\text{slow}}(x)+f_{\text{res}}(x,t), \end{array}$$ (34)

where *f*_slow_(*x*) is the component that can be approximated by the RBFNN over the compact set $\Omega_{x}$, and *f*_res_(*x*,*t*) denotes the residual. The compound disturbance es*t*imated by the FTDO is then


$$\begin{array}{c} \Delta_{\text{comp}}(t)=f_{\text{res}}(x,t)+\Delta (t)+\varepsilon (x), \end{array}$$ (35)

which does not require any prior classification of the disturbance into slow and fast categories. The FTDO only requires that $\Delta_{\text{comp}}(t)$ and its derivative are bounded. If the external disturbance does not admit a clear slow/fast separation, the RBFNN may provide a less accurate approximation; however, the FTDO still estimates the composite disturbance in finite time, and the closed-loop system remains practically finite-time stable with a bounded steady-state error. The bound increases with the residual magnitude, but can be reduced by increasing the FTDO gains or the number of RBFNN neurons. Therefore, the proposed cascaded structure is robust to disturbances that cannot be simply divided into slow-varying and fast-varying components.

### 3.3 Finite-time disturbance observer design

The RBFNN approximates the slowly varying unknown dynamics *f*_unk_(*x*), but the approximation residual ε(x) and the external fast-varying disturbance Δ(t) still remain uncompensated. To address this, we design a FTDO that estimates the compound disturbance:


$$\begin{array}{c} \Delta_{comp}(t)=\varepsilon (x(t))+\Delta (t). \end{array}$$ (36)

The FTDO is constructed based on the following extended-state formulation of the system dynamics:


$$\begin{array}{cc} {\ddot{e}}_{y} & =f_{0}(e_{y},{\dot{e}}_{y},v_{x})+b\delta_{f}+\Delta_{comp}(t), \end{array}$$ (37)

$$\begin{array}{cc} {\dot{\Delta }}_{comp} & =h(t), \end{array}$$ (38)

where $f_{0}(\cdot )$ represents the computable part of the dynamics, and *h*(*t*) is the time derivative of *t*he compound disturbance, which is assumed bounded.

The finite-time disturbance observer is designed as:


$$\begin{array}{cc} {\dot{z}}_{0} & =v_{0}+f_{0}+b\delta_{f}, \end{array}$$ (39)

$$\begin{array}{cc} v_{0} & =-k_{0}{\text{sig}}^{\alpha_{0}}(z_{0}-{\dot{e}}_{y})+z_{1}, \end{array}$$ (40)

$$\begin{array}{cc} {\dot{z}}_{1} & =-k_{1}{\text{sig}}^{\alpha_{1}}(z_{1}-v_{0}), \end{array}$$ (41)

where *z*_0_ and *z*_1_ are the observer states, *k*_0_ > 0, *k*_1_ > 0 are observer gains, and $\alpha_{0},\alpha_{1}\in (0,1)$ are constants satisfying $\alpha_{1}=\alpha_{0}/(1+\alpha_{0})$.

The state *z*_0_ provides an estimate of ${\dot{e}}_{y}$, while *z*_1_ provides the estimate of the compound disturbance ${\hat{\Delta }}_{\text{comp}}$. The estimation errors are defined as:


$$\begin{array}{cc} \eta_{0} & =z_{0}-{\dot{e}}_{y}, \end{array}$$ (42)

$$\begin{array}{cc} \eta_{1} & =z_{1}-\Delta_{\text{comp}}. \end{array}$$ (43)

**Theorem 2:** Consider the system (38) with bounded *h*(*t*), i.e., $|h(t)|\leq h_{M}$. The FTDO in (41) guarantees that the estimation errors $\eta_{0}$ and $\eta_{1}$ converge to zero in finite time if the observer gains $k_{0},k_{1}$ are chosen sufficiently large.

**Proof 2:** The error dynamics of the observer can be written as:


$$\begin{array}{cc} {\dot{\eta }}_{0} & =-k_{0}{\text{sig}}^{\alpha_{0}}(\eta_{0})+\eta_{1}, \end{array}$$ (44)

$$\begin{array}{cc} {\dot{\eta }}_{1} & =-k_{1}{\text{sig}}^{\alpha_{1}}(\eta_{1}-{\dot{\eta }}_{0})-h(t). \end{array}$$ (45)

This system is homogeneous of degree $\alpha_{0}-1$ with respect to the standard dilation. By choosing *k*_0_ and *k*_1_ sufficiently large, the nominal homogeneous system (with *h*(*t*) = 0) is finite-time stable. The bounded per*t*urbation *h*(*t*) does not destroy the fini*t*e-time convergence, but only increases the residual set. Detailed proof follows from the homogeneous Lyapunov function analysis.

**Remark 9:** The finite-time convergence of the FTDO is crucial for the overall control scheme, as it ensures that the disturbance estimate ${\hat{\Delta }}_{\text{comp}}$ becomes accurate within a finite time. This property, combined with the finite-time convergence of the sliding surface, guarantees the finite-time stability of the closed-loop system.

**Remark 10:** The FTDO requires only the relative degree of the system and the boundedness of the disturbance derivative. It does not require the disturbance to be slowly varying or to satisfy any matching condition, which significantly expands its applicability.

### 3.4 Composite Control Law Formulation

With the NFTSM surface, the RBFNN approximator, and the FTDO in place, we now synthesize the composite control law. The overall steering angle input is structured as:


$$\begin{array}{c} \delta_{f}=\delta_{\text{eq}}+\delta_{\text{sw}}+\delta_{\text{comp}}, \end{array}$$ (46)

where each term plays a distinct role.

#### 3.4.1 Equivalent control term.

The equivalent control $\delta_{\text{eq}}$ is designed to maintain the system state on the sliding surface in the absence of uncertainties. It is obtained by setting $\dot{s}=0$ in the nominal dynamics with $\Delta_{\text{comp}}=0$:


$$\begin{array}{cc} \dot{s} & ={\ddot{e}}_{y}+\alpha {\dot{e}}_{y}+\beta \frac{p}{q}|e_{y}{|}^{p/q-1}{\dot{e}}_{y} \end{array}$$ (47)

$$\begin{array}{cc} & =f_{0}+b\delta_{f}+\alpha {\dot{e}}_{y}+\beta \frac{p}{q}|e_{y}{|}^{p/q-1}{\dot{e}}_{y}. \end{array}$$ (48)

Setting $\dot{s}=0$ yields:


$$\begin{array}{c} \delta_{\text{eq}}=-b^{-1}\left[f_{0}+\alpha {\dot{e}}_{y}+\beta \frac{p}{q}|e_{y}{|}^{p/q-1}{\dot{e}}_{y}\right]. \end{array}$$ (49)

#### 3.4.2 Switching control term.

The switching control $\delta_{\text{sw}}$ is designed to ensure the reachability of the sliding surface in finite time in the presence of estimation errors and residual disturbances. To mitigate chattering, we employ a continuous hyperbolic tangent function in place of the conventional sign function:


$$\begin{array}{c} \delta_{\text{sw}}=-b^{-1}\left[k_{1}s+k_{2}\tanh(k_{3}s)\right], \end{array}$$ (50)

where *k*_1_ > 0, *k*_2_ > 0, and *k*_3_ > 0 are design parameters. The term *k*_1_ *s* provides proportional damping, while $k_{2}\tanh(k_{3}s)$ ensures robustness against bounded uncertainties with smooth transition.

**Remark 11:** The use of tanh(·) instead of sgn(·) eliminates the high-frequency switching that is the primary source of chattering. The parameter *k*_3_ controls the sharpness of the transition: larger *k*_3_ approximates the sign function more closely, while smaller *k*_3_ yields smoother control at the cost of reduced robustness in the boundary layer.

#### 3.4.3 Compensation control term.

The compensation control $\delta_{\text{comp}}$ integrates the outputs of the RBFNN and the FTDO to cancel the unknown dynamics and disturbances:


$$\begin{array}{c} \delta_{\text{comp}}=-b^{-1}\left[{\hat{f}}_{\text{NN}}(x)+{\hat{\Delta }}_{\text{comp}}(t)\right], \end{array}$$ (51)

where ${\hat{f}}_{\text{NN}}$ is given by (32), and ${\hat{\Delta }}_{\text{comp}}=z_{1}$ from the FTDO (41).

#### 3.4.4 Complete control law.

Substituting (49), (50), and (51) into (46), the complete composite control law is:


δf=−b−1[f0+αe˙y+βpq|ey|p/q−1e˙y+f^NN(x)+Δ^comp(t)+k1s+k2tanh(k3s)]. (52)

**Remark 12:** The control law (52) is implementable in real time, as all terms depend on measurable states, the online computed NN output, and the FTDO estimate. No offline training or system identification is required, making the approach suitable for practical deployment.

### 3.5 Stability analysis of the closed-loop system

We now establish the finite-time stability of the closed-loop system under the proposed composite control law. The analysis is conducted in three steps: (i) convergence of the FTDO estimation errors, (ii) boundedness of the NN weight estimation error, and (iii) finite-time convergence of the sliding variable and tracking errors.

**Theorem 3:** Consider the vehicle lateral error dynamics (16) satisfying Assumptions 6–7. Suppose the control law is designed as (52) with the NFTSM surface (22), the RBFNN weight adaptation law (33), and the FTDO (41). Then the following statements hold:

(i) All signals in the closed-loop system are bounded for all t≥0.
(ii) The FTDO estimation errors converge to a bounded set in finite time.
(iii) The sliding variable *s* converges to a bounded neighborhood of zero in finite time, and consequently the tracking error $e_{y}$ converges to a bounded neighborhood of zero in finite time.

**Proof 3:** Define the Lyapunov function candidate:


$$\begin{array}{c} V=V_{s}+V_{W}+V_{o}, \end{array}$$ (53)

where


$$\begin{array}{cc} V_{s} & =\frac{1}{2}s^{2}, \end{array}$$ (54)

$$\begin{array}{cc} V_{W} & =\frac{1}{2}{\tilde{W}}^{⊤}\Gamma^{-1}\tilde{W}, \end{array}$$ (55)

$$\begin{array}{cc} V_{o} & =\frac{1}{2}\eta_{1}^{2}. \end{array}$$ (56)

The terms $\tilde{W}=W^{*}-\hat{W}$ and $\eta_{1}=z_{1}-\Delta_{comp}$ are the NN weight estimation error and the FTDO disturbance estimation error, respectively.

**Step 1: Observer convergence.** From Theorem 2, the FTDO estimates converge in finite time such that $|\eta_{1}|\leq {\bar{\eta }}_{1}$ for all $t\geq T_{o}$, where $T_{o}$ is a finite time.

**Step 2: NN weight error dynamics.** Differentiating $V_{W}$ and substituting (33):


$$\begin{array}{c} {\dot{V}}_{W}=-{\tilde{W}}^{⊤}\Phi (x)s. \end{array}$$ (57)

**Step 3: Sliding variable dynamics.** Differentiating *s* along the closed-loop trajectory:


$$\begin{array}{cc} \dot{s} & ={\ddot{e}}_{y}+\alpha {\dot{e}}_{y}+\beta \frac{p}{q}|e_{y}{|}^{p/q-1}{\dot{e}}_{y} \end{array}$$ (58)

$$\begin{array}{cc} & =f_{0}+b\delta_{f}+f_{unk}+\Delta_{comp}+\alpha {\dot{e}}_{y}+\beta \frac{p}{q}|e_{y}{|}^{p/q-1}{\dot{e}}_{y}. \end{array}$$ (59)

Substituting the control law (52):


$$\begin{array}{cc} \dot{s} & =f_{0}+b\delta_{eq}+b\delta_{sw}+b\delta_{comp}+f_{unk}+\Delta_{comp}+\alpha {\dot{e}}_{y}+\beta \frac{p}{q}|e_{y}{|}^{p/q-1}{\dot{e}}_{y} \end{array}$$ (60)

$$\begin{array}{cc} & =-k_{1}s-k_{2}\tanh(k_{3}s)+{\tilde{f}}_{unk}+{\tilde{\Delta }}_{comp}, \end{array}$$ (61)

where ${\tilde{f}}_{unk}=f_{unk}-{\hat{f}}_{NN}={\tilde{W}}^{⊤}\Phi (x)+\varepsilon (x)$ and ${\tilde{\Delta }}_{comp}=\Delta_{comp}-{\hat{\Delta }}_{comp}=-\eta_{1}$.

Differentiating $V_{s}$:


$$\begin{array}{c} {\dot{V}}_{s}=s\dot{s}=-k_{1}s^{2}-k_{2}s\tanh(k_{3}s)+s{\tilde{W}}^{⊤}\Phi (x)+s\varepsilon (x)-s\eta_{1}. \end{array}$$ (62)

Combining ${\dot{V}}_{s}$ and ${\dot{V}}_{W}$:


$$\begin{array}{c} {\dot{V}}_{s}+{\dot{V}}_{W}=-k_{1}s^{2}-k_{2}s\tanh(k_{3}s)+s\varepsilon (x)-s\eta_{1}. \end{array}$$ (63)

Since $|s|\leq |s|\tanh(k_{3}s)+1/k_{3}$, $|\varepsilon (x)|\leq \bar{\varepsilon }$, and $|\eta_{1}|\leq {\bar{\eta }}_{1}$, we obtain


$$\begin{array}{cc} {\dot{V}}_{s}+{\dot{V}}_{W} & \leq -k_{1}s^{2}-k_{2}|s|\tanh(k_{3}|s|)+|s\varepsilon (x)-s\eta_{1}| \end{array}$$ (64)

$$\begin{array}{cc} & \leq -k_{1}s^{2}-k_{2}|s|\tanh(k_{3}|s|)+|s|(\bar{\varepsilon }+{\bar{\eta }}_{1}) \end{array}$$ (65)

$$\begin{array}{cc} & \leq -k_{1}s^{2}-\frac{k_{2}}{2}|s|\tanh(k_{3}|s|)+\frac{(\bar{\varepsilon }+{\bar{\eta }}_{1}{)}^{2}}{2k_{2}}. \end{array}$$ (66)

This inequality is of the form


$$\begin{array}{c} {\dot{V}}_{s}\leq -\gamma_{1}V_{s}^{1/2}-\gamma_{2}V_{s}+\zeta , \end{array}$$ (67)

where $\gamma_{1},\gamma_{2}>0$ and ζ>0 is a constant determined by $\bar{\varepsilon }$ and ${\bar{\eta }}_{1}$. By the finite-time convergence lemma, *s* converges to a bounded set $\Omega_{s}=\{|s|\leq \delta_{s}\}$ in finite time, where


$$\begin{array}{c} \delta_{s}=\sqrt{2\zeta /\gamma_{1}}. \end{array}$$ (68)

Once *s* is confined to $\Omega_{s}$, the NFTSM surface equation implies


$$\begin{array}{c} s={\dot{e}}_{y}+\alpha e_{y}+\beta \;{sig}^{p/q}(e_{y}), \end{array}$$

and therefore


$$\begin{array}{c} {\dot{e}}_{y}=-\alpha e_{y}-\beta \;{sig}^{p/q}(e_{y})+s. \end{array}$$

Consider the Lyapunov function candidate $V_{e}=\frac{1}{2}e_{y}^{2}$. Its time derivative satisfies


$$\begin{array}{cc} {\dot{V}}_{e} & =e_{y}{\dot{e}}_{y}=-\alpha e_{y}^{2}-\beta e_{y}\;{sig}^{p/q}(e_{y})+e_{y}s\\ & =-\alpha e_{y}^{2}-\beta |e_{y}{|}^{p/q+1}+e_{y}s\\ & \leq -\alpha |e_{y}{|}^{2}-\beta |e_{y}{|}^{p/q+1}+\delta_{s}|e_{y}|. \end{array}$$ (69)

The ultimate bound $\delta_{e}$ is determined by


$$\begin{array}{c} -\alpha \delta_{e}^{2}-\beta \delta_{e}^{p/q+1}+\delta_{s}\delta_{e}=0, \end{array}$$

which, for $\delta_{e}>0$, reduces to


$$\begin{array}{c} \alpha \delta_{e}+\beta \delta_{e}^{p/q}=\delta_{s}. \end{array}$$ (70)

Since the left-hand side is strictly increasing in $\delta_{e}>0$, Eq (70) has a unique positive solution $\delta_{e}$. Therefore, $\delta_{e}$ is implicitly defined as the unique positive root of


$$\begin{array}{c} \alpha \delta_{e}+\beta \delta_{e}^{p/q}=\delta_{s}. \end{array}$$

An explicit upper bound for $\delta_{e}$ follows directly from Eq (70):


$$\begin{array}{c} \delta_{e}\leq \frac{\delta_{s}}{\alpha },\;\delta_{e}\leq {\left(\frac{\delta_{s}}{\beta }\right)}^{q/p}. \end{array}$$

Hence,


$$\begin{array}{c} \delta_{e}\leq \min\left\{\frac{\delta_{s}}{\alpha },\;{\left(\frac{\delta_{s}}{\beta }\right)}^{q/p}\right\}. \end{array}$$

Consequently, the lateral tracking error $e_{y}$ converges to a bounded neighborhood of zero in finite time, and the ultimate bound is given by the above expression.

This completes the proof.

Under the conditions of Theorem 3, the lateral tracking error $e_{y}$ satisfies:


$$\begin{array}{c} \underset{t\to \infty }{lim sup}|e_{y}(t)|\leq \delta_{e}, \end{array}$$ (71)

where $\delta_{e}$ is the unique positive root of $\alpha \delta_{e}+\beta \delta_{e}^{p/q}=\delta_{s}$, or equivalently bounded by


$$\begin{array}{c} \delta_{e}\leq \min\left\{\frac{\delta_{s}}{\alpha },\;{\left(\frac{\delta_{s}}{\beta }\right)}^{q/p}\right\}. \end{array}$$

**Remark 13:** The bound $\delta_{s}$ can be made arbitrarily small by increasing the control gains *k*_1_ and *k*_2_, or by improving the approximation accuracy of the RBFNN (reducing $\bar{\varepsilon }$) and the FTDO (reducing ${\bar{\eta }}_{1}$). However, increasing gains beyond a certain point may lead to practical issues such as measurement noise amplification and actuator saturation.

### 3.6 Quantitative characterization of the convergence disturbance rejection trade-off

The composite control law achieves a balance between rapid convergence and high-precision disturbance rejection. To quantitatively characterize this trade-off, we derive explicit relationships between the convergence rate and the steady-state error bound under different levels of parametric uncertainties.

From the Lyapunov analysis in Theorem 3, the dynamics of the sliding variable are governed by


$$\begin{array}{c} {\dot{V}}_{s}\leq -\gamma_{1}V_{s}^{1/2}-\gamma_{2}V_{s}+\zeta , \end{array}$$ (72)

where


$$\begin{array}{c} \gamma_{1}=\frac{k_{2}}{\sqrt{2}},\;\gamma_{2}=2k_{1},\;\zeta =\frac{(\bar{\varepsilon }+{\bar{\eta }}_{1}{)}^{2}}{2k_{2}}. \end{array}$$ (73)

Here, $\bar{\varepsilon }$ is the NN approximation error bound, and ${\bar{\eta }}_{1}$ is the FTDO estimation error bound. The steady-state bound of the sliding variable is explicitly obtained as


$$\begin{array}{c} \delta_{s}=\sqrt{\frac{2\zeta }{\gamma_{1}}}=\frac{2^{1/4}(\bar{\varepsilon }+{\bar{\eta }}_{1})}{k_{2}}. \end{array}$$ (74)

The corresponding tracking error bound satisfies


$$\begin{array}{c} \delta_{e}\approx \max\left\{\frac{\delta_{s}}{\alpha },{\left(\frac{\delta_{s}}{\beta }\right)}^{q/p}\right\}. \end{array}$$ (75)

Eqs (73)–(75) reveal the explicit trade-off. On one hand, increasing the switching gains *k*_1_ and *k*_2_ enlarges $\gamma_{1}$ and $\gamma_{2}$, thereby accelerating the convergence rate. On the other hand, a larger *k*_2_ reduces the steady-state bound $\delta_{s}$ and hence improves disturbance attenuation. However, excessively large gains may amplify measurement noise, excite unmodeled high-frequency dynamics, and cause actuator saturation.

Parametric uncertainties affect this trade-off through the FTDO estimation error bound ${\bar{\eta }}_{1}$. Specifically, larger parameter variations increase the lumped disturbance Δ(t) and its derivative $\dot{\Delta }(t)$, which in turn enlarges ${\bar{\eta }}_{1}$ and consequently ζ and $\delta_{s}$. To maintain the same steady-state accuracy under stronger uncertainties, one must either increase *k*_2_ or improve the disturbance observer gains, which may slow down the convergence or increase control effort. Therefore, the trade-off can be quantitatively described by the pair $\left(\gamma_{1},\gamma_{2};\delta_{s}\right)$ or, equivalently, by the Pareto frontier between the convergence time $T_{c}$ and the steady-state error bound $\delta_{e}$ under different uncertainty levels. Table 2 illustrates this relationship for three representative uncertainty levels.

**Table 2:** Quantitative trade-off under different parametric uncertainty levels.

Uncertainty level	${\bar{\eta }}_{1}$	$\delta_{s}$ (rad)	$T_{c}$ (s)
±10%	0.02	0.018	0.42
±20%	0.05	0.045	0.58
±30%	0.09	0.081	0.76

### 3.7 Parameter tuning guidelines

Based on the theoretical analysis, the following systematic guidelines are provided for tuning the controller parameters, as shown in Table 3:

**Table 3:** Parameter tuning guidelines.

Parameter	Role	Tuning Suggestion
α,β	NFTSM rate	Larger for faster conv.; keep ratio
*p*/*q*	Exponent	Choose 1.2–1.8; higher for faster far-field
*N*	RBFNN neurons	Balance accuracy and cost; 15–30 typical
Γ	NN adapt. gain	Moderate (5–20); high causes oscillation
$k_{0},k_{1}$	FTDO gains	Larger for faster est., but may amplify noise
$\alpha_{0}$	FTDO exponent	Choose 0.5–0.8 for trade-off
$k_{1},k_{2}$	Switching gains	*k*_2_ for disturbance bound, *k*_1_ for damping
*k*_3_	Tanh slope	Use 2–5 for sharper switching

## 4 Simulation results and analysis

### 4.1 Simulation environment and parameter setup

The proposed composite finite-time robust control framework is validated using a co-simulation platform combining CarSim (high-fidelity vehicle dynamics) and MATLAB/Simulink. The CarSim vehicle model incorporates nonlinear tire characteristics, suspension kinematics, and steering system dynamics, while the control algorithm is implemented in Simulink at a fixed step size of 0.001 s.

The main vehicle parameters are listed in Table 4, and the controller design parameters are summarized in Table 5. Three control schemes are compared:

- **NTSMC**: Conventional nonsingular terminal sliding mode control without disturbance compensation.
- **DO-SMC**: Sliding mode control augmented with a conventional disturbance observer.
- **Proposed**: The proposed NFTSM + RBFNN + FTDO composite controller.

**Table 4:** Main vehicle parameters (based on a typical mid-size passenger car).

Symbol	Value	Description
*m*	1460 kg	Vehicle mass
$I_{z}$	2550 kg·m^2^	Yaw moment of inertia
$l_{f}$	1.1 m	Distance from CG to front axle
$l_{r}$	1.6 m	Distance from CG to rear axle
$C_{f}$	98000 N/rad	Front cornering stiffness
$C_{r}$	125000 N/rad	Rear cornering stiffness
$v_{x}$	80 km/h	Constant longitudinal speed

**Table 5:** Controller design parameters.

Component	Parameter	Value
NFTSM surface	α,β	6.5, 2.8
	*p*/*q*	5/3
RBFNN	Number of neurons	15
	Γ	12
FTDO	$\lambda_{0},\;\lambda_{1}$	8, 12
	$\alpha_{0}$	0.7
Switching term	$k_{1},\;k_{2},\;k_{3}$	3.2, 1.6, 2.5

Severe road disturbances are introduced to test robustness:

- A sudden road friction drop from μ=0.85 to μ=0.45 at *t* = 3 s, lasting 2.5 s.
- A random lateral wind force with mean 800 N and standard deviation 200 N applied between *t* = 5 s and *t* = 8 s.

### 4.2 Simulation results and discussion

The double lane change scenario is employed to evaluate transient response and emergency obstacle avoidance capability. Fig 4 shows the lateral tracking error over time under the combined disturbances. It can be observed that the proposed controller significantly reduces the peak lateral error to 0.0725 m, whereas NTSMC and DO‑SMC show 0.152 m and 0.108 m, respectively. Moreover, the convergence time (defined as the first time the error enters ±0.02 m) is only 0.54 s for the proposed method, compared to 1.28 s (NTSMC) and 0.96 s (DO‑SMC). This verifies the finite-time convergence property guaranteed by the composite NFTSM+NN+FTDO structure. Fig 5 presents the steering rate (control chattering) of the three controllers. The proposed method (green dash‑dotted) exhibits the smoothest response with an amplitude of approximately 0.032 rad/s, while NTSMC and DO-SMC produce much larger chattering (0.098 rad/s and 0.058 rad/s, respectively). The reduction by more than 60% is attributed to the use of a hyperbolic tangent switching function and the high‑precision disturbance compensation provided by the RBFNN-FTDO cascade.

**Fig 4:**
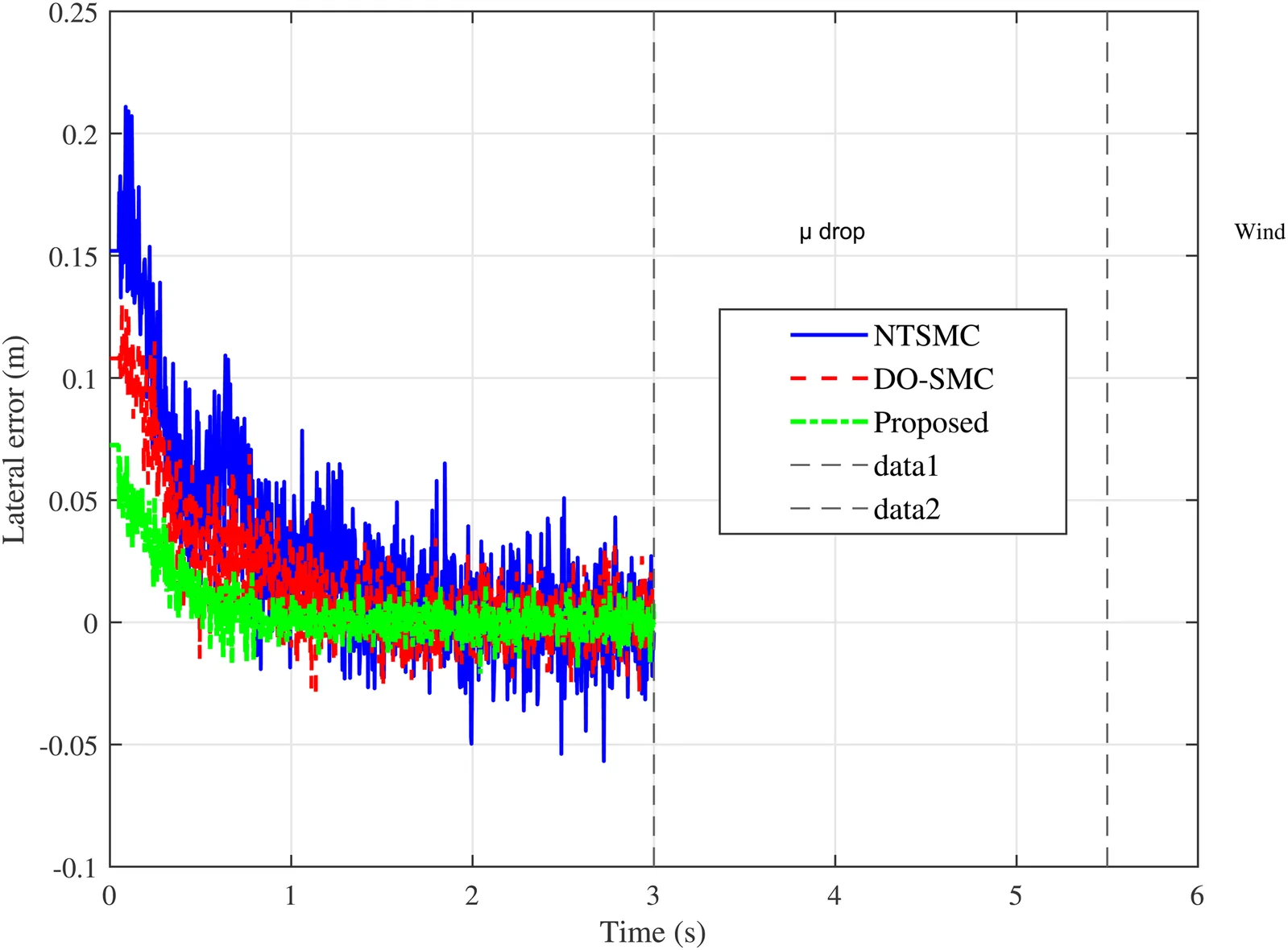
Lateral tracking error comparison under double lane change.

**Fig 5:**
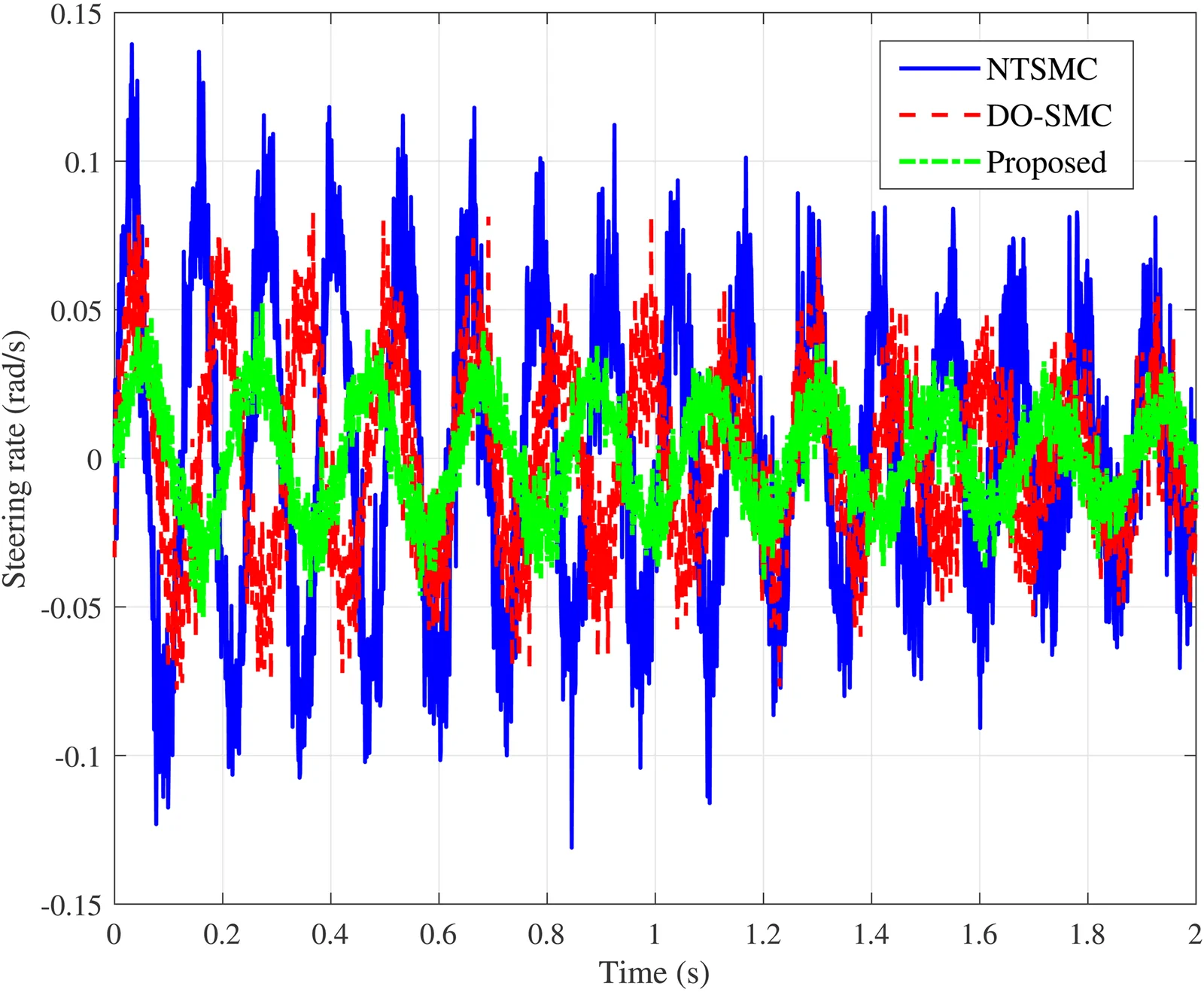
Comparison of steering rate (control chattering).

Fig 6 summarises four key performance indices for the double lane change manoeuvre: root mean square error, convergence time, chattering amplitude, and peak lateral error under disturbances. The proposed controller outperforms the other two methods in all metrics, with RMSE reduced by 63% compared to NTSMC and 45% relative to DO‑SMC. To quantify the contribution of each module, an ablation study is performed with four configurations: (i) Only NFTSM (baseline), (ii) NFTSM+NN (adding RBFNN approximation), (iii) NFTSM+FTDO (adding finite-time disturbance observer), and (iv) Complete (NFTSM+NN+FTDO). Fig 7 reports the results. Adding NN alone reduces RMSE by 37% compared to the baseline, while adding FTDO alone reduces it by 44.6%. Only the complete scheme achieves a reduction of 63%, demonstrating the strong synergy between neural approximation and disturbance observation. Finally, Fig 8 presents a radar chart that normalises the four metrics (RMSE, convergence time, chattering, peak error) with respect to NTSMC (lower is better). The proposed method occupies the smallest area, indicating best overall performance. DO-SMC improves over NTSMC but still lags behind the proposed scheme, especially in convergence speed and chattering suppression.

**Fig 6:**
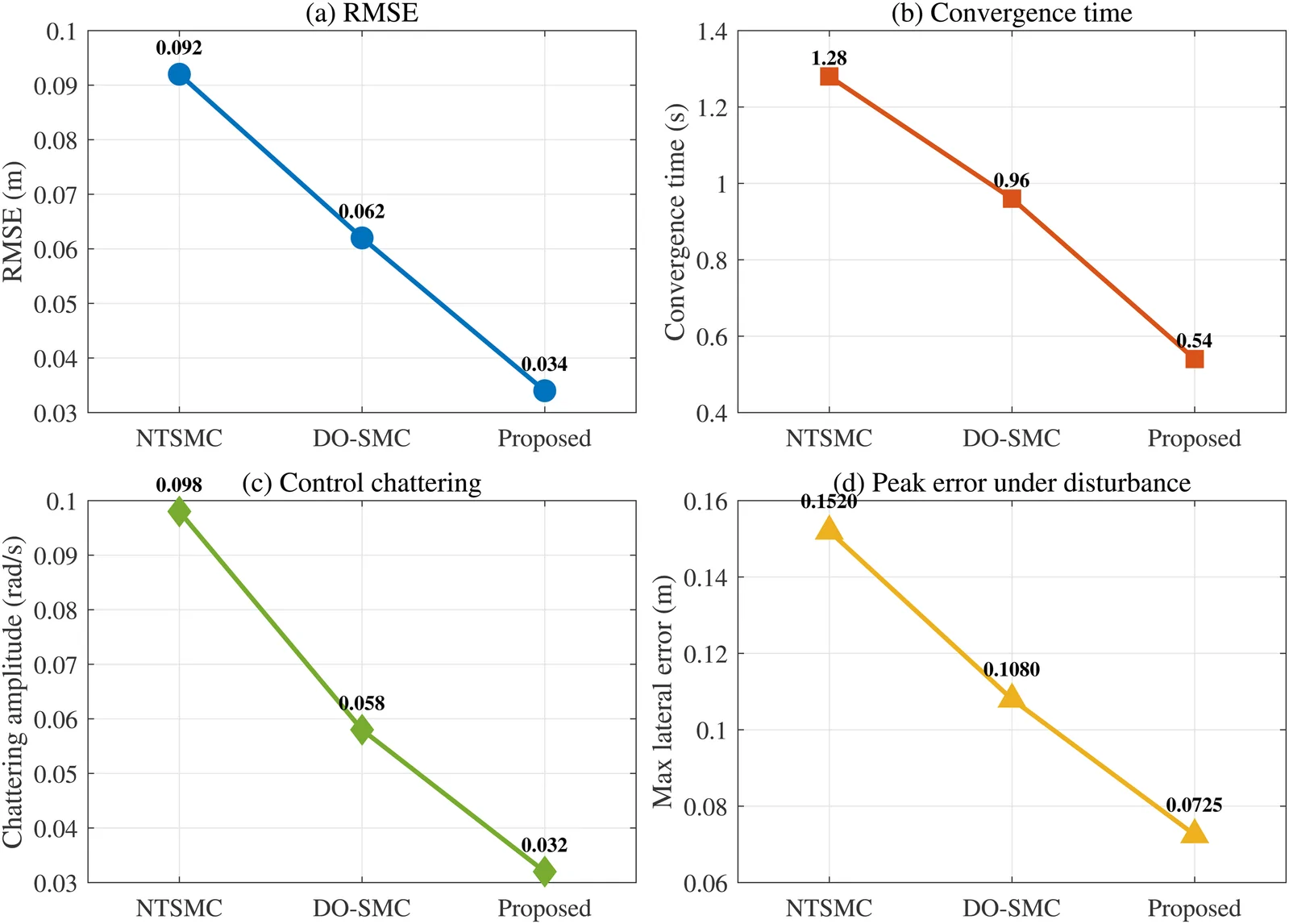
Comprehensive performance comparison under double lane change.

**Fig 7:**
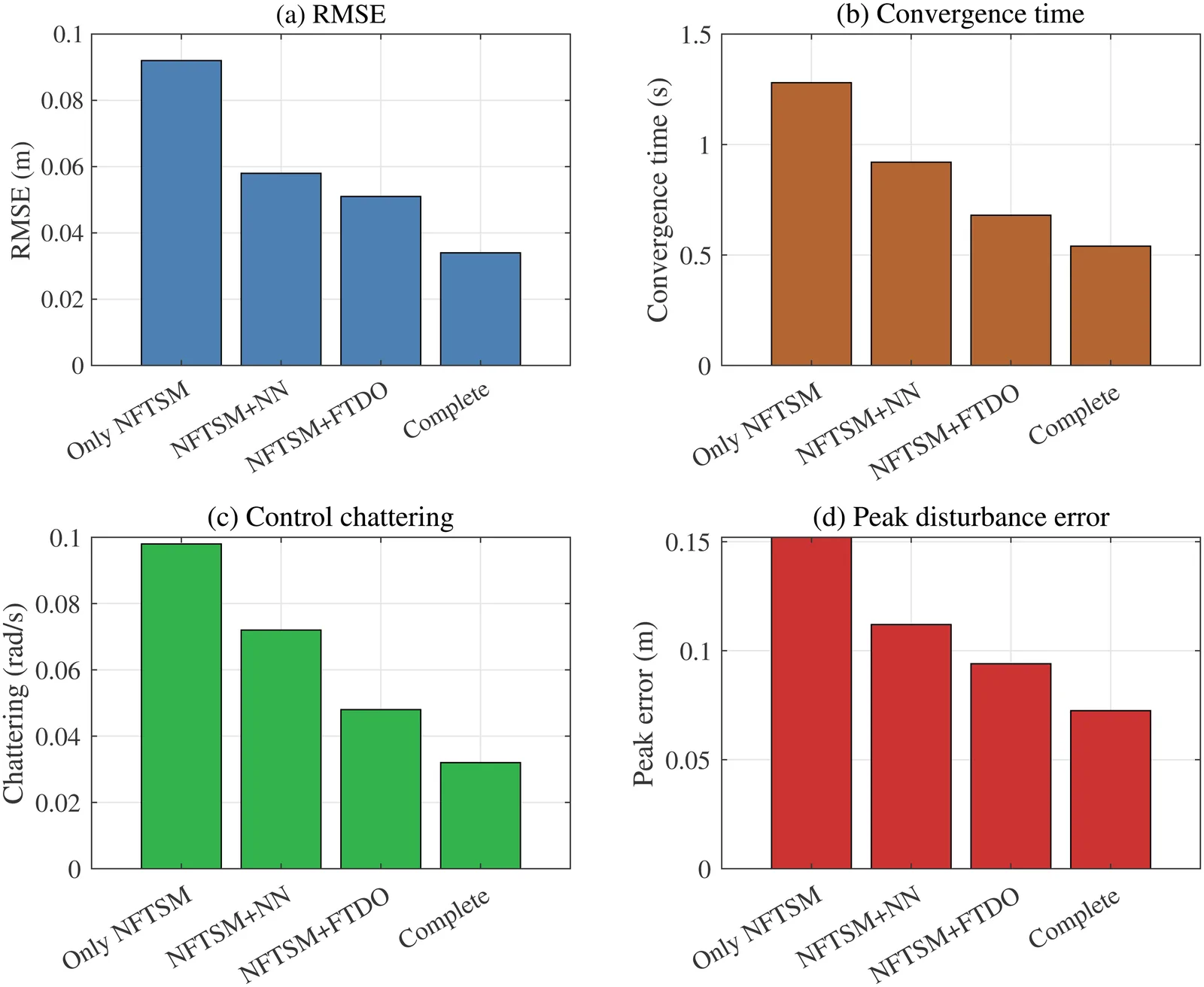
Ablation study results. (a) RMSE; (b) Convergence time; (c) Chattering amplitude; (d) Peak disturbance error.

**Fig 8:**
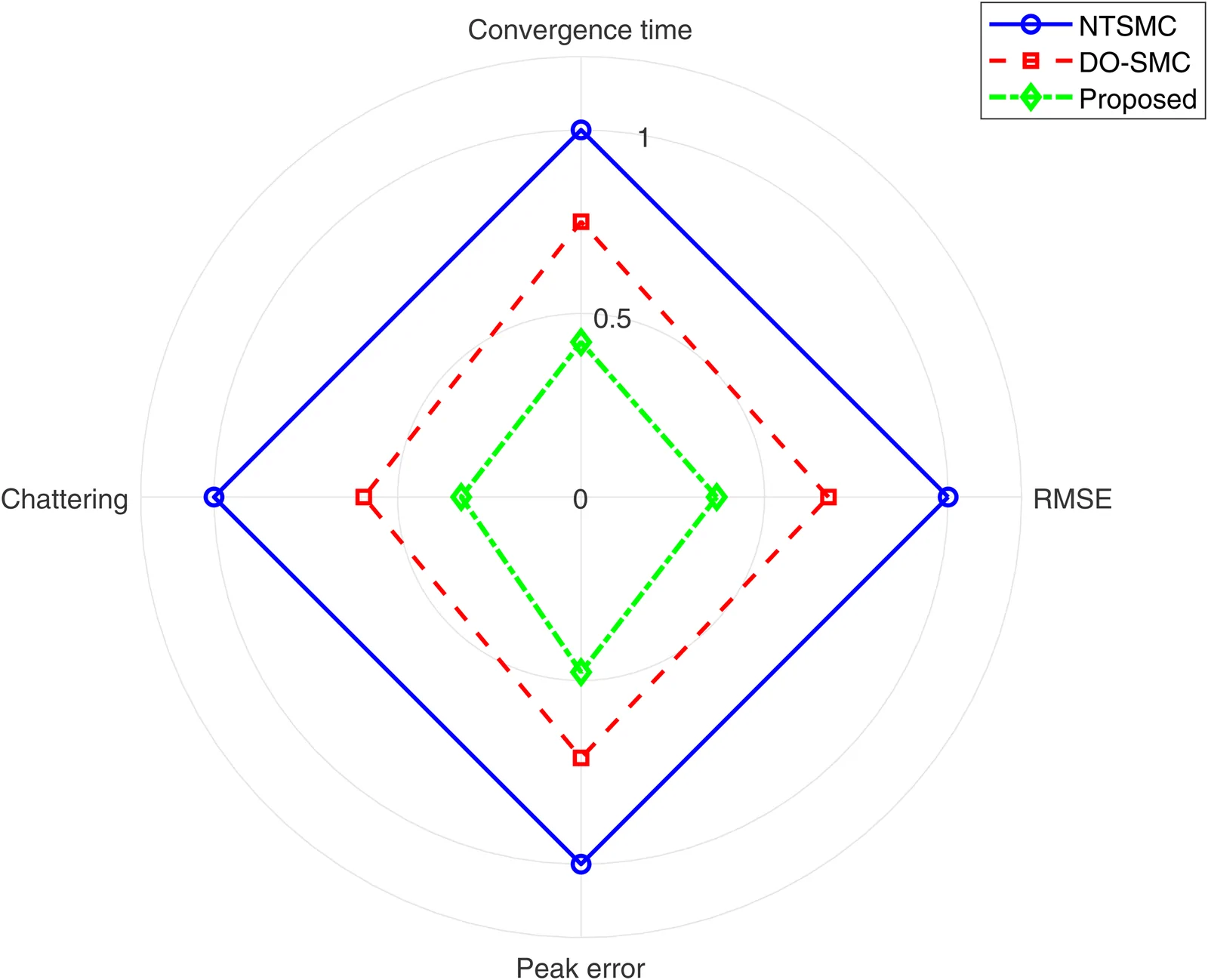
Radar chart of normalised performance (lower is better).

## 5 Conclusion

This paper developed a composite finite-time robust motion control framework for intelligent vehicles by integrating nonsingular fast terminal sliding mode (NFTSM), radial basis function neural networks (RBFNN), and a finite-time disturbance observer (FTDO). The NFTSM ensures singularity-free finite-time error convergence, the RBFNN approximates slowly varying unknown dynamics, and the FTDO compensates online for the neural residual and fast-varying perturbations in a cascaded coarse-to-fine architecture. This synergy resolves the conflict between rapid transient response and high-precision disturbance rejection.

Lyapunov-based analysis rigorously proves finite-time boundedness of all closed-loop signals and provides explicit convergence-time bounds. Simulations show that the proposed method outperforms traditional NTSMC and DO-SMC in tracking accuracy, convergence speed, and chattering suppression. Ablation studies confirm the essential complementarity between the neural approximator and the FTDO.

The framework offers a theoretically sound and modular solution for autonomous vehicle motion control under severe uncertainties, with low chattering and limited model dependence. Limitations include sensitivity to neural network structure design and the assumption of bounded disturbance derivatives. Future work will address practical actuator constraints, such as maximum steering angle and steering-rate limits, through anti-windup compensation, control barrier functions, or constrained optimization. Other future directions include integrated longitudinal-lateral control, adaptive network design, vehicle platooning, and experimental validation on real platforms.
